# Supplementary material for: Intentional abortion and its associated factors among female sex workers in Iran: Results from national bio-behavioral surveillance-2020
Source: PLoS One. 2022 Aug 29;17(8):e0273732. doi: 10.1371/journal.pone.0273732 (PMC9423624; doi:10.1371/journal.pone.0273732)
Supplement: Attachment — Submitted filename: Respose abortion 30 July.docx [file pone.0273732.s002.docx]

Author's response to decision letter for (EJMR-D-21-01338): “Intentional abortion and its associated factors among female sex workers in Iran: Results from National Bio-Behavioral Surveillance-2020.”

July 30, 2022

Dear Editor,

We appreciate you and the reviewers of the “PloS One” journal for your precious time reviewing our paper and providing valuable comments. Your helpful and insightful comments led to possible improvements in the current version. The authors have carefully considered the statements and tried their best to address every one of them. We hope the manuscript, after careful revisions, meets your high standards. The authors welcome further constructive comments if any.

Below we provide the point-by-point responses highlighted in red.

Best Regards,

Yousef Moradi, PhD

Yousefmoradi211@yahoo.com

Social Determinants of Health Research Center, Research Institute for Health Development, Kurdistan University of Medical Sciences, Sanandaj 6617713446, Iran.

Review Comments to the Author

We've checked your submission and before we can proceed, we need you to address the following issues:

1. Thank you for updating your Data Availability statement to:

"The data supporting this study's findings cannot be shared publicly because of ethical restrictions involving patient information. Data are available on request from the Social Determinants of Health Research Center, Research Institute for Health Development, Kurdistan University of Medical Sciences after appropriate protocol submission to the institution’s office of Human Research Ethics Committee. (contact via sdhkurdistan@gmail.com) for researchers who meet the criteria for access to confidential data."

Can you please clarify who is the recipient of the provided contact email "sdhkurdistan@gmail.com" and what their role is at the Social Determinants of Health Research Center, Research Institute for Health Development, Kurdistan University of Medical Sciences?

Response:

Thank you so much. The data supporting this study's findings cannot be shared publicly because of ethical restrictions involving patient information. Data are available on request from the Social Determinants of Health Research Center, Research Institute for Health Development, Kurdistan University of Medical Sciences after appropriate protocol submission to the institution’s office of Human Research Ethics Committee. (Contact via [sdhkurdistan@gmail.com](mailto:sdhkurdistan@gmail.com) ) for researchers who meet the criteria for access to confidential data.

The Social Determinant of Health Research Center began its work in 2012. During two years this center published over 100 articles in international and national journals, and it has carried out many research projects in the public health issues. Often, these projects were funded by WHO, UNAIDS, UNDP, and Iranian Ministry of Health. This center has a Research Council Members for deciding about projects and related their data. This Research Council Members included several experts and researchers related to public health issues (https://muk.ac.ir/Page?pageId=8714). This group will be decided to publish national and subnational data. **The Dr. Bakhtiar Piroozi is head of center and he responsible to receive and response all requests.**
